# Global patterns and correlates in the emergence of antimicrobial resistance in humans

**DOI:** 10.1098/rspb.2023.1085

**Published:** 2023-09-20

**Authors:** Emma Mendelsohn, Noam Ross, Carlos Zambrana-Torrelio, T. P. Van Boeckel, Ramanan Laxminarayan, Peter Daszak

**Affiliations:** ^1^ EcoHealth Alliance, 520 Eighth Avenue, Ste. 1200, New York, NY 1018, USA; ^2^ ETH Zurich, Rämistrasse 101, 8092 Zürich, Switzerland; ^3^ One Health Trust, 5636 Connecticut Avenue NW, PO Box 42735, DC 20015, USA; ^4^ Princeton University, NJ 08554, USA; ^5^ University of Gothenburg, Medicinaregatan 3, 413 90 Göteborg, Sweden

**Keywords:** antimicrobial resistance, global health, AMR, antibiotic resistance

## Abstract

Antimicrobial resistance (AMR) is a critical global health threat, and drivers of the emergence of novel strains of antibiotic-resistant bacteria in humans are poorly understood at the global scale. We examined correlates of AMR emergence in humans using global data on the origins of novel strains of AMR bacteria from 2006 to 2017, human and livestock antibiotic use, country economic activity and reporting bias indicators. We found that AMR emergence is positively correlated with antibiotic consumption in humans. However, the relationship between AMR emergence and antibiotic consumption in livestock is modified by gross domestic product (GDP), with only higher GDP countries showing a slight positive association, a finding that differs from previous studies on the drivers of AMR prevalence. We also found that human travel may play a role in AMR emergence, likely driving the spread of novel AMR strains into countries where they are subsequently detected for the first time. Finally, we used our model to generate a country-level map of the global distribution of predicted AMR emergence risk, and compared these findings against reported AMR emergence to identify gaps in surveillance that can be used to direct prevention and intervention policies.

## 1. Introduction

The emergence of antimicrobial resistance (AMR) is a critical global health and economic challenge. AMR bacterial strains have been associated with increased mortality, longer illnesses, medical complications in surgery, barriers to chemotherapy and higher healthcare costs [1–4]. Global human use of antibiotics has increased substantially over the past two decades, with an alarming uptick in last-resort compounds that are administered when other treatments fail [5]. Rates of human use of antibiotics correlate with resistance rates in pathogenic bacteria at multiple scales and locations [6–9]. Combating AMR has become a priority for governments (e.g. the United States National Action Plan for Combating Antibiotic-Resistant Bacteria; UK Five-Year National Action Plan for Tackling Antimicrobial Resistance; Australia's National Antimicrobial Resistance Strategy) and intergovernmental organizations (e.g. Tripartite-Plus Alliance on AMR, Food and Agriculture Organization, World Organisation for Animal Health, United Nations Environment Programme and World Health Organization), multi-lateral development banks and financing facilities (e.g. World Bank) and global One Health initiatives (e.g. One Health High-Level Expert Panel, OHHLEP).

Antibiotics are used routinely in animal husbandry to prevent and treat bacterial diseases and they have been used as growth promoters to expedite weight gain, a practice that has been banned in recent years in many countries [10,11]. Antibiotic use in livestock—which vastly exceeds their use in humans—has enabled intensive husbandry, and is projected to increase by 67% globally, and to nearly double in Brazil, Russia, India, China and South Africa by 2030 [11,12]. Resistance genes, AMR bacterial strains and plasmids including some of human clinical relevance, such as Methicillin-resistant *Staphylococcus aureus*, have been reported from livestock, wildlife and environmental samples [11,13–16]. These findings have led to policy efforts to reduce antibiotic use in livestock [3,4].

To our knowledge, there are no published analyses on the relative roles of human and livestock consumption of antibiotics in driving the emergence of novel strains of antibiotic-resistant bacteria in human clinical cases. In the current study, we use a database that we assembled of global AMR emergence events, containing 1611 records of the first clinical reports of novel bacterial resistance over 11 years from 2006 to 2017, to examine global patterns in the emergence of new AMR strains in humans [17]. We model how observed AMR emergence events are correlated with human and livestock consumption of antibiotics; human population size and mobility (migrant population and tourism); economic activity (gross domestic product (GDP) and healthcare expenditure); antibiotic exports as a proxy for production; and biomedical surveillance effort. We then use these correlations to produce predictive models of the global distribution of AMR emergence risk.

A key challenge in interpreting global patterns of AMR emergence is variation in AMR surveillance and reporting. Underreporting in lower-income countries is a persistent problem in AMR datasets [2,18], and may be particularly important given that many lower-income countries are most affected by resistant infections (e.g. malaria, tuberculosis, neonatal sepsis) [3,19,20] and are experiencing the greatest increases in consumption of antibiotics in humans and livestock [11,18]. In this study, we apply methods used in our previous work analysing the emergence of zoonoses [21,22] to correct for underlying biases in reporting novel emergence by using quantitative metrics of biomedical surveillance.

## 2. Results

Our AMR emergence database contains 1611 records of first clinical reports of novel bacterial resistance occurring in 60 countries from 2006 to 2017, extracted from biomedical literature. The USA had the greatest number of reported events (*n* = 132), followed by India (*n* = 127), China (*n* = 120), Canada (*n* = 98) and Japan (*n* = 75). For more details on the structure and methods to build the database, see [17].

We modelled the rate of reported population-corrected AMR emergence as a function of human antibiotic consumption, livestock antibiotic consumption, GDP, healthcare expenditure, antibiotic exports, inbound tourism, migrant population and measures of reporting and publication bias. To account for the high frequency of zeros in the AMR data (133 of 193 countries with no reported events), we used a Poisson-hurdle model with a conditional (Poisson) and a zero-inflated (logistic) component, conceptually representing the AMR emergence rate and probability of zero reporting, respectively. Our model explained 70% (s.d. = 3.3%) of country-level variance in *per capita* AMR emergence rates. To interpret the model results, we report incidence rate ratios (IRR) for count-based predictions of AMR emergence rates and odds ratios (OR) for the zero-inflated probability. We also report Bayesian 95% credible intervals (CI) on the effects and we interpret a predictor as associated with the outcome when the CI does not include one.

Human *per capita* antibiotic consumption was positively associated with AMR emergence rates (IRR = 1.04 per defined daily dose *per capita* (DDD *per capita*); 95%CI = 1.00–1.08) (table 1). For a country in which AMR emergence is expected (i.e. non-zero prediction), a 36% (±50%) greater than average AMR emergence rate is expected at twice the average human antibiotic consumption (mean human antibiotic consumption = 7.3 DDD *per capita*), with all other variables held at average.

**Table 1.** Incidence rate ratios (Poisson, conditional model) and odds ratios (logistic, zero-inflated) and 95% credible intervals (CI) of predictors in the main model. Asterisks indicate that the variable is associated with the outcome (i.e. 95% CI does not include one); blue shading represents a positive association and orange shading represents a negative association. AB, antibiotic; DDD, daily dose *per capita*.

model	predictor	estimate
conditional (Poisson)	GDP (log US$ *per capita*)	7.1 (1.8–22)*
	livestock AB consumption & GDP interaction	1.4 (1–1.8)*
	tourism—inbound (log *per capita*)	1.3 (1.2–1.5)*
	ProMed mentions (log *per capita*)	1.2 (0.88–1.5)
	migrant population (log *per capita*)	1.1 (0.92–1.3)
	human AB consumption (DDD)	1 (1–1.1)*
	English spoken (yes/no)	1 (0.76–1.4)
	health expenditure (% GDP)	0.9 (0.86–0.95)*
	AB exports (log dollars *per capita*)	0.9 (0.84–0.95)*
	publication bias index (log *per capita*)	0.89 (0.75–1.1)
	livestock AB consumption (log kg *per capita*)	0.019 (0.0017–0.51)*
zero-inflated (logistic)	ProMed mentions (log *per capita*)	3.8 (1.3–13)*
	English spoken (yes/no)	1.3 (0.35–5.2)
	publication bias index (log *per capita*)	0.84 (0.4–1.8)
	population (log)	0.43 (0.24–0.69)*
	GDP (log dollars *per capita*)	0.18 (0.051–0.51)*

*Per capita* GDP was positively associated with AMR emergence rates (IRR = 7.1 per log-dollars *per capita*; 95%CI = 1.8–22), while negatively associated with a country's zero-reporting probability (i.e. lower GDP countries were less likely to report any emergence events; OR = 0.18 per log-dollars *per capita*; 95%CI = 0.051–0.51).

**Figure 1.**
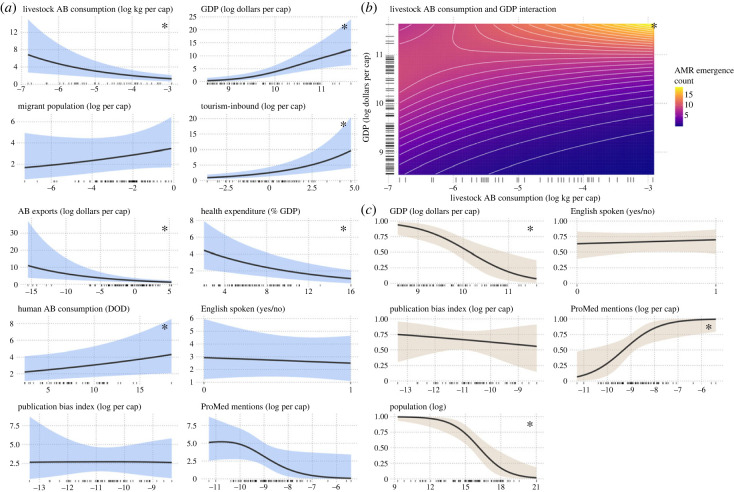
Conditional effects for each model variable, showing the posterior mean of the response distribution with all other variables held at average. (*a*) From the conditional (Poisson) model, mean predicted AMR emergence counts relative to non-interaction terms and (*b*) the interaction between livestock antibiotic consumption and GDP. (*c*) From the zero-inflated (logistic) model, probability of zero AMR emergence reporting relative to reporting bias predictors. In (*a*) and (*c*), ribbons represent the 95% probability distribution on the mean, and the solid line is the median prediction. In (*b*), contours represent the median surface prediction. In all plots, rug ticks show raw values (missing value imputation not represented) and asterisks indicate that the variable is associated with the outcome (i.e. the 95% CI of the IRR or OR does not include one). AB, antibiotic.

Because initial data exploration found that the relationship between livestock antibiotic consumption and AMR emergence rates differed between low- and high-income countries, our model included an interaction term for livestock antibiotic consumption and country GDP. The main (non-interaction) effect of livestock antibiotic consumption was negatively associated with AMR emergence rates (IRR = 0.019 per log of kg antibiotics consumed by livestock *per* human *capita*; 95%CI = 0.0017–0.51). However, the interaction between livestock antibiotic consumption and GDP, both normalized to human population size, was a positive predictor of AMR emergence rates, increasing the effect of livestock antibiotic consumption in correlation with increasing GDP (IRR = 1.4; 95%CI = 1.0–1.8; table 1). Overall, at average GDP ($17 000 *per capita*), a doubling of livestock antibiotic consumption predicts a 26% (±31%) decrease in AMR emergence rates. At low GDP ($5700 *per capita*, the 5th percentile among countries), doubling livestock consumption predicts a 55% (±24%) decrease in AMR emergence rates. The relationship between livestock antibiotic consumption and AMR emergence rates becomes positive at a GDP value of approximately $46 000 *per capita*, and at high GDPs ($57 000 *per capita* [95th percentile GDP]), doubling consumption predicts a 39% (±56%) increase in AMR emergence rates (figure 1).

Inbound tourism volume per country, normalized to human population size, was positively associated with AMR emergence rates (IRR = 1.3 per log of inbound tourists *per capita*; 95%CI = 1.2–1.5). For a country in which AMR emergence is expected (i.e. non-zero prediction), a 22% (±44%) greater than average AMR emergence rate is expected at twice the average inbound tourism (mean inbound tourism *per capita* = 1.3), with all other variables held at average. Migrant population, normalized to human population size, was not associated with the outcome. The dollar value of antibiotic exports, normalized to human population size, was inversely associated with AMR emergence rates (IRR = 0.90 per log of antibiotic exports *per capita*; 95%CI = 0.84–0.95). Healthcare expenditure as a percentage of GDP was also inversely associated with AMR emergence rates (IRR = 0.90 per cent of GDP; 95%CI = 0.84–0.95).

We compared different variables as proxies for reporting bias in AMR reports. Human population size was negatively associated with a country's zero-reporting probability (i.e. countries with lower populations were less likely to report any emergence event; OR = 0.43 *per capita*; 95%CI = 0.24–0.69). Conversely, the number of *per capita* ProMED reports related to a country (ProMED mentions) was positively associated with a country's zero-reporting probability (i.e. countries with fewer ProMED mentions were more likely to report an emergence event; OR = 3.8 per log of ProMED mentions *per capita*; 95%CI = 1.3–13), and ProMED mentions were not associated with emergence rates. Neither English-speaking in a country nor the publication bias index were associated with reporting of AMR emergence events or with AMR emergence rates.

Due to lack of data on human and livestock antibiotic consumption for many, especially low-income countries (electronic supplementary material, table S1), we used model-imputed values for these correlates. To test robustness of results, we evaluated results under four imputation scenarios: (i) no imputation of antibiotic consumption (*n* = 36); (ii) imputation of either human or livestock antibiotic consumption (*n* = 74); (iii) imputation of human and livestock antibiotic consumption for countries within GDP range of countries with complete human and animal antimicrobial consumption data (*n* = 95); and (iv) full imputation (*n* = 193). While IRR and OR differed among the models, the overall direction of effects was largely consistent, and interpretation did not vary drastically between models (electronic supplementary material, table S2). Human antibiotic consumption was positively associated with AMR emergence rates in all models except for the no-imputation model (electronic supplementary material, table S2 *Model 1*), which included only 36 of 68 available human antibiotic consumption data points. The effects of livestock antibiotic consumption, GDP, and their interaction were the same across models except for the full imputation model (electronic supplementary material, table S2 *Model 4*), which imputed 152 of 193 of livestock antibiotic consumption data points. Here, we report results from the third model—imputation of human and livestock antibiotic consumption for countries within the GDP range of countries with complete human and animal antimicrobial consumption data. We selected this model because it maximizes data coverage without predicting beyond the conditions of the observed data. Results for the other models are reported in the electronic supplementary material.

To ensure robustness of our results, we tested various model formulations. Given that the USA is a singular outlier in metrics such as the number of reported events, GDP, publication bias index, ProMED mentions and antibiotic exports, we conducted a separate model analysis excluding the USA. In this model, neither livestock antibiotic consumption nor the interaction of livestock antibiotic consumption with GDP were associated with the AMR emergence rates, although the direction of the effects remained the same (electronic supplementary material, table S2 *Model 3.1*).

In a separate model, we replaced *per*-human-*capita* livestock antibiotic consumption with per-livestock biomass antibiotic consumption and found that per-livestock biomass antibiotic consumption was not associated with AMR emergence rates while livestock population size on its own was inversely associated with emergence rates (electronic supplementary material, table S2 *Model 3.2*). Finally, following Jones *et al*. [22], we repeated the analysis on a subset of emergence data representing the first temporal emergence of unique drug–pathogen combinations in a human population (i.e. including only the first country in which resistance of a pathogen to a drug is observed). Results from this model were largely consistent with the main model (electronic supplementary material, table S2 *Model 3.3*).

**Figure 2.**
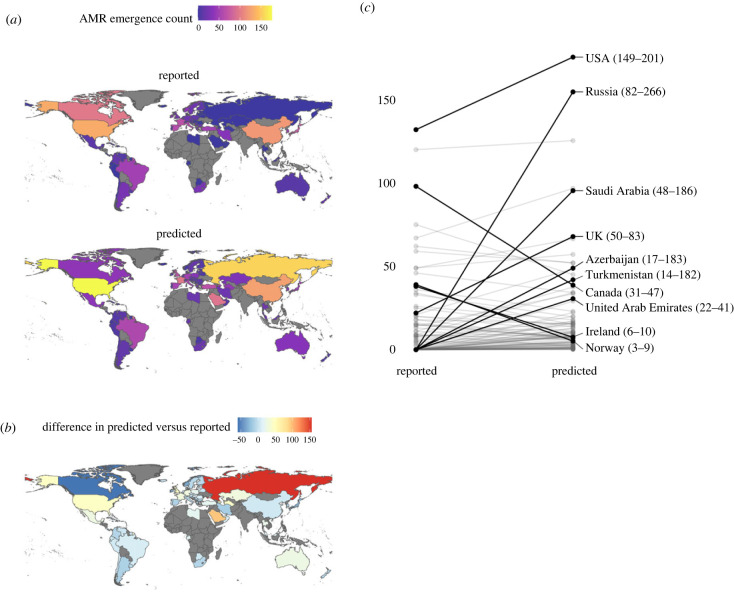
(*a*) Reported and median predicted AMR emergence event counts for the 95 countries included in our main model. (*b*) Difference between predicted and reported counts. (*c*) Ten countries with the largest absolute difference between reported and median predicted counts, with 95th percentile predicted range in parentheses.

We used our model to estimate reporting-corrected AMR emergence rates for each country (i.e. predicted rates conditional on reporting, using only the Poisson component of the hurdle model; figure 2). These results show higher predicted rates for 74% of countries, including those that have the highest counts in our database (USA, China) and in countries that previously reported few or zero events. Countries with the greatest increase in predicted AMR counts were Russia (95th percentile range = 82–266), Saudi Arabia (48–186) and Azerbaijan (17–183), all of which had zero reported events in our database.

## 3. Discussion

This paper reports the first global analysis of drivers of the emergence of AMR in humans, with efforts to correct for reporting bias and inconsistencies in data on antibiotic use. Previous studies have described the presence, prevalence of, and trends in caseloads over time for specific resistant strains [7,9,14,23–25]. Byarugaba [19] and Bonn [26] described increasing rates of resistant isolates and clinical antimicrobial-resistant infections in developing and developed nations, respectively, and identified potential risk factors. Bell *et al*. [8] conducted a meta-analysis showing a correlation between antibiotic use in humans and AMR rates measured at various resolutions, from bacterial isolates to the country level. Van Boeckel *et al*. [11] analysed AMR in livestock, finding increased rates of resistance in common indicator pathogens in animals correlating with increased antimicrobial use for animal husbandry (we used data from this study for our livestock antibiotic consumption variable). Other studies have analysed patterns of use or sale of antibiotics for human or livestock use. For example, Goossens *et al.* [6] described rates of human antibiotic usage in European countries, and more recently Klein *et al.* [18] analysed the trends and drivers of human antibiotic consumption in 76 countries (we used this latter source for human antibiotic consumption data in our current analysis). Van Boeckel *et al*. [12] generated a global map of antimicrobial usage in livestock and projected a global rise in overall usage in the coming decade.

In this study, we analysed the origins and likely drivers of global emergence of AMR, using records of first clinical reports of unique bacterial–drug cases from 2006 to 2017 [17], datasets of antimicrobial drug sales for human and livestock use, and published strategies for dealing with reporting bias. We found that human use of antimicrobials is positively correlated with the origins of AMR events in people, and that this scales with population-corrected DDD *per capita*. Previous analyses of AMR trends have modelled the presence or prevalence of specific resistant strains and provided evidence that antibiotic use in people directly contributes to AMR in hospitals and clinics, communities and countries [6–9,27]. However, this correlation has not previously been demonstrated for the emergence of resistance on a global scale, controlling for reporting biases, and over a broad swath of AMR pathogen/drug combinations. Another prior study assessed how socio-economic and demographic factors correlate with an index of AMR in 103 countries and found that human antimicrobial drug use was not correlated with resistance [28]. The current study, however, analyses the drivers of the first known clinical cases of a novel AMR emergence, whereas [28] analysed the level of resistance to several drug classes in three pathogens encountered in clinics in a country. Our analysis is consistent with the findings of [28] that AMR in a country is likely driven in part by contagion—the spread of AMR after its emergence—and that this occurs independently to the degree of antibiotic consumption. Together, these papers provide a more detailed explanation of what drives the origins, spread and impact of AMR, and are therefore of value in developing policy to control each aspect of emergence.

We found that the highest GDP countries have a slight positive association between antibiotic consumption for animal husbandry and the origins of new AMR strains in people, and lower GDP countries have a neutral or negative association. In our robustness analysis—where the USA was removed from the dataset as an outlier in multiple metrics including GDP and reporting effort—we found this interaction was no longer a predictor of AMR emergence (i.e. the 95% CI did not include one), but the direction of the effect was the same. Under a separate formulation in which we normalized livestock antibiotic consumption to livestock biomass instead of human population size, no association between livestock antibiotic consumption and AMR emergence was observed. These findings of a weak relationship between livestock antibiotic consumption and AMR emergence may reflect that most AMR emergence events are to recently introduced antibiotics (imipenem, ciprofloxacin and ceftazidime were the most represented in our database), which may correlate better with human antibiotic usage, rather than general use of antibiotics in livestock. Introduction of newer antibiotics may occur at a slower pace in livestock than in humans, due in part to the higher price of newer antibiotics. This rate of introduction of new antibiotics to livestock may be modified by GDP, which could explain the presence of a slight positive association between livestock antibiotic use and AMR emergence at the highest GDPs. Nonetheless, given the limited number of data points for livestock antibiotic consumption (*n* = 41), additional data collection is needed to better understand the relationship between animal husbandry and AMR emergence, and our findings suggest that this relationship may be complex or mediated by other factors.

Other research has demonstrated that antibiotic use for animal husbandry is a significant public health threat in contributing to the spread of specific existing resistance strains in humans [24,29]. Further, a meta-analysis found evidence from 13 studies that interventions to reduce animal use of antimicrobials led to a reduction in the presence of antibiotic-resistant bacteria in humans, particularly those with direct contact with food-producing animals [30]. We conclude that, while our analysis indicates that human use of antibiotics is likely more important for human AMR emergence than animal use, further research is needed to better understand the patterns of transmission of AMR strains among livestock and people [24,29,31]. We hypothesize that dense populations of livestock may act as maintenance or amplifying hosts for known AMR strains, a scenario similar to the role of intermediate livestock hosts in the emergence of novel zoonoses such as Nipah virus disease and MERS [32].

Environmental contamination by antibiotics has been increasingly linked to the emergence and spread of AMR [13,33]. In our analysis, we assumed that countries with greater exports (and we assume production) of antibiotics would have higher environmental contamination. However, we found that countries with higher antibiotic exports had lower rates of AMR emergence (with some variability in the consistency of this effect across model scenarios; see electronic supplementary material, table S2), suggesting that environmental contamination is not a significant driver of novel strain emergence or that antibiotic exports are a poor proxy for environmental contamination. This does not exclude the possibility of environmental contamination being a factor in maintaining or spreading AMR strains once they have emerged.

To assess if the emergence of a novel strain is caused by the spread of infection (bacterium or gene transmission) into a country rather than its *de facto* evolution and origin, we included measures of human population movement in our model. Inbound tourism, normalized to population size, was a positive predictor of AMR emergence rates, while inbound migration, normalized to population size, was not. These results suggest that first emergences in a country may be driven, in part, by the spread of existing resistant strains from other countries. We repeated the analysis on a subset of emergence data representing first global appearances of unique drug–pathogen combinations (i.e. including only the first country in which resistance of a pathogen to a drug is observed). This analysis did not alter our findings related to tourism and migration, suggesting that mechanisms of spread (e.g. gene transfer) in addition to mutation may also drive first global emergences.

More developed public infrastructure and higher metrics of good governance inversely correlate with AMR rates [28]. In our study, we used data on GDP *per capita* and healthcare expenditure as proxies for the ability of countries to control AMR, identify cases and manage consumption patterns. GDP *per capita* was positively associated with AMR emergence rates. Healthcare expenditure as a percentage of GDP was inversely associated with AMR emergence rates, but this relationship was no longer present when we removed the USA from the dataset. These findings likely reflect the fact that our outcome measure is not the level of resistance seen in clinics in a country (e.g. prevalence, incidence, occurrence of known or novel AMR strains), but rather the number of novel AMR strains originating in a country. The latter may be more strongly correlated with human antibiotic drug use as a driver of the evolution and emergence of novel strains, while the former is linked to ability to control these strains once they have emerged.

In previous work, we analysed global trends and identified predictive hotspots of emerging infectious diseases [22] and emerging zoonoses [21] by correcting for underlying biases in reporting of novel emergence. In the current study, we accounted for country-level surveillance and reporting effort using a Poisson-hurdle model approach, which separately estimates the probability of a country reporting zero events and the *per capita* rate of AMR emergence conditional on reporting. Our model included several predictors as proxies for reporting bias: country GDP, human population size, use of English language in a country (as the database was limited to English-language literature), number of ProMED mentions, and a publication bias index produced previously [21]. We found that human population size and GDP were negatively associated with zero-reporting (i.e. positively associated with AMR emergence reporting), while the number of ProMED mentions was positively associated with zero-reporting. We found no association between English language in a country or the publication bias index and AMR emergence reporting. In addition, our hurdle-model approach allowed us to estimate reporting-corrected AMR emergence counts for each country in our main model dataset (*n* = 95). We found the greatest increase in predicted counts in Russia, Saudi Arabia and Azerbaijan, all of which had zero reported events. These findings point to significant reporting gaps in these countries and the need to apply surveillance beyond the relatively limited number of countries where surveillance currently occurs.

There are several limitations to this study. First, it analyses trends in novel AMR strains reported in the literature from 2006 to 2017 against data collected on antibiotic usage in humans and animals from specific years (2014 and 2010, respectively). Variation in antibiotic usage in the 11 years of AMR reporting could cause confounding if countries have reduced or modified antimicrobial use (e.g. banning antibiotics as growth promoters in livestock). Second, it uses published data on novel AMR strains. While we included several measures of reporting and publication bias, the changes in interest or capacity to diagnose and identify AMR over this period may have varied among countries irrespective of economic capacity, due to trends in research fields. Third, data availability of some of the correlates is skewed to richer countries. Livestock antibiotic consumption data, estimated from country-reported antibiotic sales from livestock [11], is especially sparse (available for 41 countries) and biased towards developed economies. To avoid over-estimating associations, we limited our dataset to countries that have a GDP within the range of GDPs of countries that report both human and livestock consumption ($5166 *per capita* [Thailand]–$111 542 *per capita* [Luxembourg]), and therefore our results should not be extrapolated beyond this range. Finally, it is important to emphasize that the relationships discussed in this paper are associative, and causality can only be hypothesized through this type of global analysis. Further work on the mechanisms of what drives the origin of new strains, and what drives their maintenance, amplification and spread is urgently needed.

## 4. Methods

### (a) Data and variable selection

We used AMR emergence data from the database described in [17] (https://zenodo.org/record/4924992), which contains records of first clinical reports of unique bacterial–drug AMR detections from 1998 to 2017, drawn from scientific literature and disease surveillance reports. We filtered the database for events starting in 2006 and later, as database coverage prior to 2006 is limited to disease surveillance reports. To perform analyses at the country level, we summed the count of emergence events by country. This approach allows the same drug–bacteria combination to be represented in multiple country counts. As part of our robustness analysis (below), we also ran the model using first reported global emergences as an alternative outcome (i.e. each drug–bacteria combination reported only once).

We generated hypotheses that AMR emergence would be influenced by human and livestock consumption of antibiotics, antibiotic production, importation via inbound travel, expenditures on healthcare and economic capacity. We then identified datasets that served as proxy variables for each of these factors. Predictor variables are from multiple sources, listed in electronic supplementary material, table S1. We identified straightforward measures for human and livestock consumption of antibiotics, human population size and healthcare expenditure. We used migrant population and inbound tourism as proxies for inbound human travel, and we used antibiotic exports as a proxy for production. In addition, we included five variables representing reporting bias based on previous experience: human population size, GDP, the number of ProMED reports from a country, and a publication bias index based on references to place names in biomedical publications from the PubMed Central Open-Access Subset [21]. We also included whether English language was spoken in the country because our database is based on English language publications. Similar approaches of accounting for reporting bias have been used for a variety of global-scale disease detection studies [34–36].

Prior to modelling, highly skewed continuous variables were natural log-transformed. We examined data for collinearity via Spearman rank coefficients and used this as guidance for normalizing some variables to GDP or human population size, as indicated in the *measurement units* field of electronic supplementary material, table S1. After these transformations, Spearman rank coefficients were less than 0.7 for all variable pairs. Livestock antibiotic consumption was normalized to human population size, rather than livestock population size, as we are interested in the potential contribution of antibiotic use in agriculture to antibiotic exposure and AMR emergence in humans. In our robustness analysis (§4c below), we ran an alternative version of the model with livestock antibiotic consumption normalized to livestock biomass. In addition, we included an interaction term for livestock antibiotic consumption and country GDP, as we noted that the association between livestock antibiotic use and AMR emergence differed between low- and high-income countries during exploratory data analysis.

### (b) Missing data handling

We limited the total number of countries in the dataset to those that have human population size and GDP data available (*n* = 193). As shown in electronic supplementary material, table S1, data availability was not consistent across other variables. We inferred zeros for missing values for the AMR emergence field, and one half the minimum value for the publication bias index, ProMED mentions and antibiotic export fields.

The following remaining variables were unavailable for some countries, with a distinct bias of missing data in low-income countries: human antibiotic consumption, livestock antibiotic consumption, health expenditure and inbound tourism. We imputed missing values for these variables, using four approaches to check for robustness:

(1) *No imputation of antibiotic consumption*—Dataset limited to countries with values for both human *and* animal antimicrobial consumption (*n* = 36).
(2) *Imputation of either human or livestock antibiotic consumption*—Dataset includes countries with values for human *and*/*or* animal antibiotic consumption (*n* = 74).
(3) *Imputation of human and livestock antibiotic consumption for countries within GDP range*—Dataset includes countries that are missing both human and livestock antibiotic consumption *if* the country has a GDP within the range of GDPs of countries from model 1 ($5166 *per* *capita* [Thailand] – $111 542 *per* *capita* [Luxembourg]), which have both human *and* animal antimicrobial consumption data (*n* = 95).
(4) *Full imputation*—Includes all countries in the dataset (*n* = 193).

We used a multivariate imputation by chained equations (MICE) algorithm with classification and regression trees (CARTs) to model missing values based on the available data [37]. CARTs are commonly used for imputation for their robustness against outliers and ability to handle multicollinearity and skewed distributions [37]. For each variable, we generated 30 imputations, each with 40 iterations. We visually examined diagnostic plots to confirm convergence. We included two additional variables—antibiotic imports and livestock biomass—in the MICE routine to better estimate missing consumption data. We did not include these variables in the model itself, however, as consumption was a more direct proxy for the hypothesized mechanism of antibiotic exposure.

### (c) Robustness scenarios

We tested several alternative formulations of our model to determine robustness of results. We tested several alternative formulations of our model to determine robustness of results, using model 3 for missing data handling (see above; *n* = 95). First, because the USA is a singular outlier in the number of reported events, GDP, publication bias index, ProMED mentions and antibiotic sales, we ran a model with the USA removed. Second, we used an alternative scaling of livestock antibiotic consumption, replacing *per*-human-*capita* livestock antibiotic consumption with per-livestock biomass antibiotic consumption. In this formulation, we also included livestock biomass, defined as the total mass of cattle, pig and chicken populations within a country [12], as a separate feature to allow disaggregation of the effects of livestock antibiotic consumption and livestock biomass. Finally, we tested an alternative model formulation with the outcome variable as the first *global* emergence of antibiotic strains, rather than first national emergence. In this formulation, a drug–bacteria combination is counted only in the single country in which it first emerged in our dataset.

### (d) Model approach

We used a Poisson-hurdle model, in which the response is a function of two components: a zero-inflated logistic component representing the probability of a country reporting zero events, and a Poisson component of the number of AMR emergence events in the period, conditional on observed reporting. For the logistic equation, we included our five reporting bias variables (human population size, GDP, English language spoken, ProMED mentions and publication bias index). All variables were included in the Poisson component, with human population size treated as an offset variable to estimate AMR emergence as a *per capita* rate.

We took a Bayesian approach to estimate model parameters, using a No-U-Turn Hamiltonian Monte Carlo implemented in Stan [39] and assuming a wide Student's *t*-distribution (*ν* = 3, *μ* = 0, *σ* = 10) for all coefficient priors [38,39]. We used four Markov chains with 2000 iterations per chain to fit the model on each of the multiple imputed datasets. Posterior samples were then combined across all imputed datasets to generate posterior distributions.

We visually examined Markov chain trace plots and used effective sample convergence statistics ({\hat{R}} convergence less than 1.05) to confirm convergence across chains [40]. We compared posterior predictions to the empirical distribution of AMR events with density overlay plots and interval plots. In addition, we compared the proportion of zeros in the posterior predictions with the empirical proportion to confirm that hurdle model accurately captured the excess zeros in the dataset (electronic supplementary material, figure S1).

### (e) Model predictions

We calculated reporting-corrected AMR emergence count predictions for all countries in our main model dataset (*n* = 95) using only the Poisson component of the model, assuming a zero-inflated (logistic) probability of 0 for all countries (i.e. assuming full reporting by all countries). By removing the logistic equation from the hurdle model, we were able to estimate AMR emergence counts corrected for excess zeros due to underreporting. We used a sample of 500 *β* coefficients generated from our model for each variable to be able to produce median and 95th percentile count estimates for each country.

### (f) Software and reproducibility

Data analysis was performed in R version 4.2.1 [41], using the tidyverse framework for data manipulation [42] and the drake package for workflow design [43]. We used the mice package [44] for the MICE imputation routine and the brms package [45], built on the Stan language [39] for Bayesian model fitting. Visual model diagnostics were generated with the bayesplot package [46].

## Data Availability

All code and data used in this project are available for download at https://github.com/ecohealthalliance/amr-analysis and on Zenodo (https://zenodo.org/record/7051951). An earlier version of this manuscript can be found on medRxiv (https://doi.org/10.1101/2022.09.29.22280519). A summary of data sources and additional figures are provided in electronic supplementary material [47].
